# Synthesis and evaluation of bifunctional sGC regulators

**DOI:** 10.1186/2050-6511-14-S1-P15

**Published:** 2013-08-29

**Authors:** Mikolaj Chrominski, Dorota Gryko, Iraida Sharina, Emil Martin

**Affiliations:** 1 Institute of Organic Chemistry, Polish Academy of Sciences, Warsaw, Poland; 2 University of Texas Houston Medical School, Department of Internal Medicine, Division of Cardiology, Houston, Texas, USA

## Background

**Figure 1:**
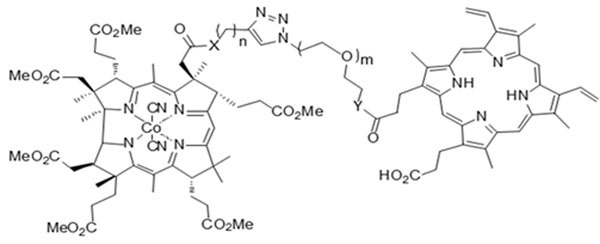
Cobinamides analog conjugated to a protoporphyrin IX derivative.

Our previous studies identified dicyanocobinamide as a novel sGC regulator that targets the catalytic region and synergistically enhances activation of sGC by NO-independent regulators. As proof-of-concept study, we designed and synthesized a set of bifunctional sGC regulators, which consist of cobinamide analog conjugated to a protoporphyrin IX derivative through linkers of varying length and composition (Figure 1).

## Results

The length and composition of the linker was proved to be crucial for sGC activation. Our results indicate that only hybrid molecules containing a 13-16 atom chain linker benefited from synergistic engagement of both heme-binding region and catalytic domain. The hybrid compounds containing linkers connected through an ester bond were not only more stable, but also were more effective than those connected though an amide. Hybrids with shorter or longer linkers, or with different linker composition, were much less potent and were no more active than the cobyrinic acid component alone. The most effective compound was conjugated through an ester bond, contained a 13 atom chain linker and a triazole group close to the cobyrinate moiety. This compound displayed more than 60 fold activation of purified sGC.

## Conclusion

These studies reinforce the concept that cobinamides can be used as co-stimulators of sGC activity and propose the principle for multiple ligand sGC regulators. Structural insights obtained from these studies lay the foundation for the creation of future bifunctional sGC regulators containing cobinamide derivatives. The library of generated cobyrinic acid building blocks adapted for “click chemistry” can be used for generation of more potent hybrids, e.g. hybrids with other heme-targeting sGC regulators.

